# MicroRNA-27a-5p regulation by promoter methylation and *MYC* signaling in prostate carcinogenesis

**DOI:** 10.1038/s41419-017-0241-y

**Published:** 2018-02-07

**Authors:** Daniela Barros-Silva, Pedro Costa-Pinheiro, Henrique Duarte, Elsa Joana Sousa, Adriane Feijó Evangelista, Inês Graça, Isa Carneiro, Ana Teresa Martins, Jorge Oliveira, André L. Carvalho, Márcia M. Marques, Rui Henrique, Carmen Jerónimo

**Affiliations:** 1 Cancer Biology and Epigenetics Group, IPO Porto Research Center (CI-IPOP), Portuguese Oncology Institute of Porto (IPO Porto), Porto, Portugal; 2 Master in Oncology, Institute of Biomedical Sciences Abel Salazar—University of Porto (ICBAS-UP), Porto, Portugal; 3 Molecular Oncology Research Center, Barretos Cancer Hospital, Barretos, São Paulo Brazil; 4 Department of Urology, Portuguese Oncology Institute of Porto (IPO Porto), Rua Dr. António Bernardino de Almeida, 4200-072 Porto, Portugal; 5 Barretos School of Health Sciences, Barretos, São Paulo Brazil; 6 Department of Pathology, Portuguese Oncology Institute of Porto (IPO Porto), Porto, Portugal; 7 Department of Pathology and Molecular Immunology, Institute of Biomedical Sciences Abel Salazar (ICBAS)—University of Porto, Porto, Portugal

## Abstract

Upregulation of *MYC* and miRNAs deregulation are common in prostate cancer (PCa). Overactive *MYC* may cause miRNAs’ expression deregulation through transcriptional and post-transcriptional mechanisms and epigenetic alterations are also involved in miRNAs dysregulation. Herein, we aimed to elucidate the role of regulatory network between *MYC* and miRNAs in prostate carcinogenesis. *MYC* expression was found upregulated in PCa cases and matched precursor lesions. MicroRNA’s microarray analysis of PCa samples with opposed *MYC* levels identified miRNAs significantly overexpressed in high-*MYC* PCa. However, validation of miR-27a-5p in primary prostate tissues disclosed downregulation in PCa, instead, correlating with aberrant promoter methylation. In a series of castration-resistant PCa (CRPC) cases, miR-27a-5p was upregulated, along with promoter hypomethylation. *MYC* and miR-27a-5p expression levels in LNCaP and PC3 cells mirrored those observed in hormone-naíve PCa and CRPC, respectively. ChIP analysis showed that miR-27a-5p expression is only regulated by c-Myc in the absence of aberrant promoter methylation. MiR-27a-5p knockdown in PC3 cells promoted cell growth, whereas miRNA forced expression in LNCaP and stable *MYC*-knockdown PC3 cells attenuated the malignant phenotype, suggesting a tumor suppressive role for miR-27a-5p. Furthermore, miR-27a-5p upregulation decreased EGFR/Akt1/mTOR signaling. We concluded that miR-27a-5p is positively regulated by *MYC*, and its silencing due to aberrant promoter methylation occurs early in prostate carcinogenesis, concomitantly with loss of *MYC* regulatory activity. Our results further suggest that along PCa progression, miR-27a-5p promoter becomes hypomethylated, allowing for *MYC* to resume its regulatory activity. However, the altered cellular context averts miR-27a-5p from successfully accomplishing its tumor suppressive function at this stage of disease.

## Introduction

Prostate cancer (PCa), the most incident cancer in men worldwide^1^, is a heterogeneous disease, ranging from clinically indolent to extremely aggressive forms^2^. Increased knowledge about the biological mechanisms underlying PCa onset and progression are likely to contribute to improved clinical and therapeutic management.

Over the last years, epigenetic mechanisms’ deregulation has arisen as major player in cancer development and recent efforts have been devoted to associate microRNAs (miRNAs) deregulated expression and tumorigenesis. MiRNAs are a class of small non-coding RNAs, with approximately 22 nucleotides in length^3^, highly conserved along the evolutionary chain, with tissue and developmental stage-specific expression^4^. These molecules are responsible for negative regulation of respective mRNA targets and have been extensively implicated in several critical cellular pathways^5^. MiRNAs are often downregulated in cancer, however, although the precise mechanisms underlying this deregulation are still under scrutiny. Importantly, there is increasing evidence sustaining that miRNAs are regulated in a similar fashion to protein-coding genes^6^.

Inappropriate activation of *MYC* oncogene is a common event in several human malignancies, and 8q gain, where it is mapped, associates with worse prognosis in PCa^7^. Interestingly*, MYC* hyperactivity has been shown to promote miRNAs’ expression deregulation through transcriptional and post-transcriptional mechanisms^8^. Moreover, epigenetic modifications such as DNA methylation and histone modifications, have been also implicated miRNA’s expression regulation. Indeed, miRNAs promoter regions undergo methylation in several neoplasms^9,10^. Hence, miRNA’s may either be overexpressed through genetic mechanisms (such as gene amplification, transcription factors)^11^ or downregulated by genetic and/or epigenetic events (e.g., deletion or promoter hypermethylation, respectively)^12^. In addition, alterations in miRNA’s biogenesis machinery may also contribute to miRNA expression variations^13^.

We have previously shown that miRNAs expression was globally downregulated in PCa and that aberrant promoter methylation was the underlying mechanism in some cases^14–16^. Herein, we aimed to extend those observations, assessing the putative role of *MYC* in miRNAs’ regulation, specifically of miR-27a-5p, in prostate carcinogenesis.

## Results

### Upregulation of *MYC* in clinically localized prostate cancer tissue samples (cohort #1)

**Fig. 1:**
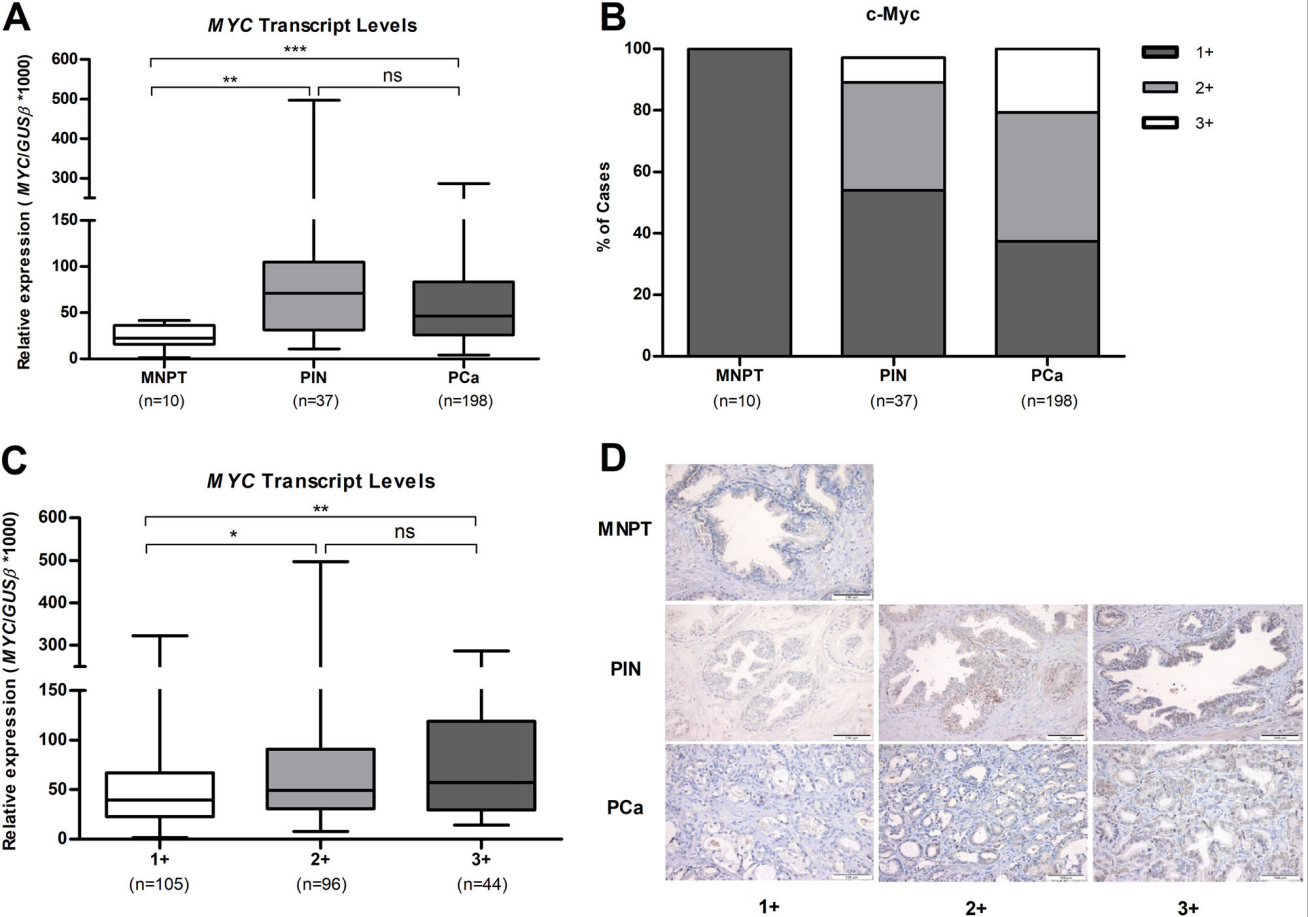
*MYC* transcript levels (**a**) and distribution of c-Myc immunostaining (**b**) in cohort #1; *MYC* transcript levels in cohort #1, grouped according to c-Myc immunostaining (**c**); Representative images of c-Myc immunostaining in cohort #1 (**d**)

*MYC* transcript and protein levels were assessed in 198 PCa (primary prostate cancer) cases, 37 PIN (prostatic intraepithelial neoplasia) lesions and 10 MNPT (morphologically normal prostate tissue) (Fig. 1a). PCa and PIN depicted significantly higher *MYC* transcript levels compared with MNPT (*P* < 0.001). Moreover, a significant increase of c-Myc protein levels was also apparent from MNPT to PCa samples (Table 1, Fig. 1b, d). Statistically significant differences were observed concerning *MYC* transcript and respective protein levels across the three groups of immunostaining scores (*P* < 0.001), and, overall, c-Myc protein levels followed the same trend (Fig. 1c). In pairwise comparisons, however, statistical significance was observed for +1 vs. +2 (*P* < 0.001) and +1 vs. +3 score groups (*P* < 0.001), but not for +2 vs. +3 scores.

**Table 1:** Immunohistochemical expression of c-Myc in a series of of PCa, PIN lesions, and MNPT

	Negative	Positive	
Clinical sample group cohort #1	1+ (≤10%) *N* (%)	2+ (10%< - ≤50%) *N* (%)	3+ (>50 %) *N* (%)
MNPT	10 (100 %)	—	—
PIN	20 (54 %)	13 (35.1 %)	4 (8.1 %)
PCa	74 (37.4 %)	83 (41.9 %)	41 (20.7 %)

Clinicopathological data from all clinical samples tested in this study are depicted in Table 2. No statistically significant differences between the two groups of patients were found for age. Furthermore, a statistically significant association was disclosed between c-Myc protein levels and some clinicopathological parameters. Somer’s *D* coefficient test revealed that higher c-Myc protein levels associated with higher serum PSA (Somer’s *D* = 0.157; *P* = 0.011) and higher GS (Somer’s *D* = 0.131; *P* = 0.044) in PCa.

**Table 2:** Clinical and pathological data of the fresh-frozen tissues included in this study

Clinicopathological data cohort #1	PCa (*n* = 198)	PIN (*n* = 37 matched with a PCa)	MNPT (*n* = 10)
Age (years), median (range)	64 [49–75]	65 [51–75]	58 [45–79]
PSA (ng/mL), median (range)	8.10 (2.66–35.50)	n.a.	n.a.
Pathological stage, *N* (%)			
pT2	110 (55.6 %)	n.a.	n.a.
pT3a	65 (32.8 %)	n.a.	n.a.
pT3b	23 (11.6 %)	n.a.	n.a.
Gleason score, *N* (%)			
<7	67 (33.8 %)	n.a.	n.a.
=7	115 (58.1 %)	n.a.	n.a.
>7	16 (8.1 %)	n.a.	n.a.

### Regulatory network between c-Myc and microRNAs

Three PCa cases with low *MYC* expression and four with high*-MYC* expression were chosen for microarray analysis. The resulting heatmap shows only miRNAs that achieved statistical significance and revealed 78 miRNAs that were overexpressed in samples with high*-MYC* content, representing possible targets of c-Myc regulation (Supplementary Fig. 1). From these, a panel of three miRNAs (miR-27a-5p, miR-570, and miR-1292) were selected for validation in a larger and independent data set. Selection of miR-27a-5p, miR-570, and miRNA-1292 was based on a critical review of published studies so that miRNAs without prior documented implication in PCa were considered for further analysis. Further validation was only accomplished for miR-27a-5p, since very low expression levels of miR-570 and miR-1292 in the clinical samples impaired the amplification reaction.

### MicroRNA-27a-5p status in clinically localized prostate cancer tissue samples (cohort #1)

**Fig. 2:**
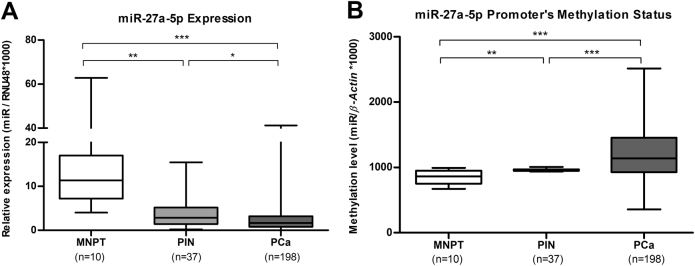
Expression levels of miRNA-27a-5p (**a**) and methylation levels of miR-27a-5p promoter (**b**) in cohort #1

Contrarily to our expectations, in the validation series (cohort #1), miRNA-27a-5p expression levels were significantly downregulated in PCa (*P < *0.001) and PIN lesions (*P < *0.01), compared with MNPT (Table 3, Fig. 2a). In an attempt to explain the previous result and since a CpG island was found at miR-27a-5p promoter region, promoter methylation status was assessed in the same cohort #1. PCa samples depicted significant higher methylation levels than PIN lesions (*P < *0.001) and MNPT (*P < *0.001) (Table 3, Fig. 2b). Moreover, there was a significant inverse correlation between miR-27a-5p promoter’s methylation and expression levels in PCa (Spearman’s rho = -0.263; *P* < 0.05). Nonetheless, in cases with miRNA promoter hypomethylation (18/198 cases), *MYC*-overexpressing PCa correlated positively with miR-27a-5p expression levels (Spearman’s rho = 0.333; *P* < 0.05) (Supplementary Fig. 2).

**Table 3:** miR-27a-5p expression and promoter methylation levels

Cohort #1	MNPT	PIN	PCa
miR-27a-5p expression Median (IQR)	11.34 (7.18–17.00)	2.88 (1.42–5.18)	1.68 (0.80–3.18)
miR-27a-5p methylation Median (IQR)	868.86 (741.32–960.97)	960.29 (949.32–971.81)	1136.28 (926.00–1448.47)

### *MYC* and miR-27a-5p in castration-resistant prostate cancer (cohort #2)

**Fig. 3:**
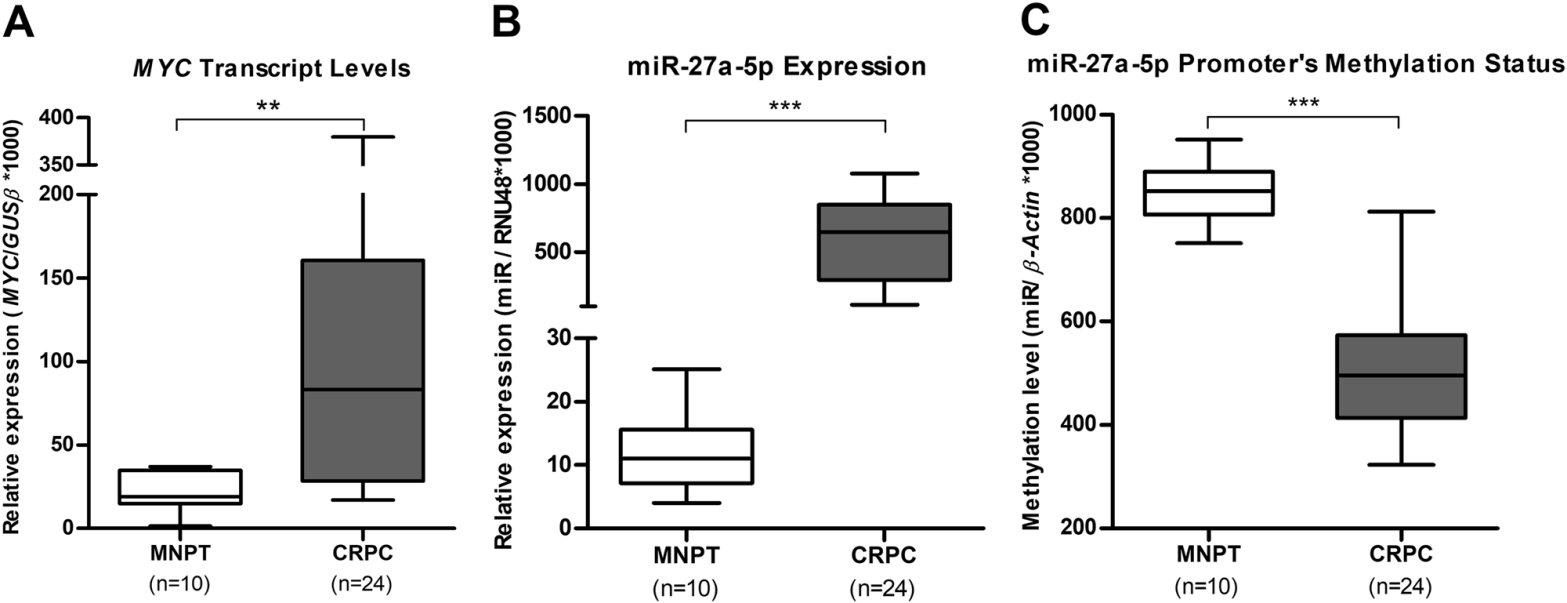
*MYC* transcript levels (**a**), expression levels of miRNA-27a-5p (**b**) and methylation levels of miR-27a-5p promoter (**c**) in cohort #2

*MYC* and miR-27a-5p status were also assessed in a second cohort of FFPE tissues consisting of castration-resistant PCa (CRPC). Clinicopathological data from cohort #2 are depicted in Table 4. No statistically significant difference between PCa and MNPT samples were found for age. A significant increase in both *MYC* (*P < *0.01) (Table 5, Fig. 3a) and miR-27a-5p (*P < *0.001) (Table 5, Fig. 3b) expression levels was found in CRPC compared to MNPT. Contrarily, miR-27a-5p promoter methylation levels in CRPC were significantly lower than those found in MNPT (*P < *0.001) (Table 5, Fig. 3c). Moreover, *MYC* overexpression correlated positively with miR-27a-5p expression levels (Spearman’s rho = 0.274; *P* < 0.05), whereas a significant inverse correlation was observed between miR-27a-5p promoter’s methylation and respective expression levels (Spearman’s rho = -0.434; *P* < 0.05).

**Table 4:** Clinical and pathological data of the FFPE tissues included in this study

Clinicopathological data cohort #2	CRPC *n*=24	MNPT *n*=10
Age (years), median (range)	66 [55–82]	58 [45–79]
PSA (ng/mL), median (range)	60.88 (1.20–360.00)	n.a.
Gleason score, *N* (%)		
<7	5 (20.8 %)	n.a.
=7	11 (45.8 %)	n.a.
>7	8 (33.3 %)	n.a.

**Table 5:** MYC and miR-27a-5p status in castration-resistant prostate cancer

Cohort #2	MNPT	CRPC
*MYC* expression median (IQR)	18.33 (14.09–34.89)	83.25 (28.49–160.52)
miR-27a-5p expression median (IQR)	10.68 (7.10–13.16)	645.87 (293.75–847.82)
miR-27a-5p methylation median (IQR)	852.35 (806.93–889.56)	495.46 (413.78–573.54)

### Characterization of *MYC* and miR-27a-5p status in PCa cell lines

**Fig. 4:**
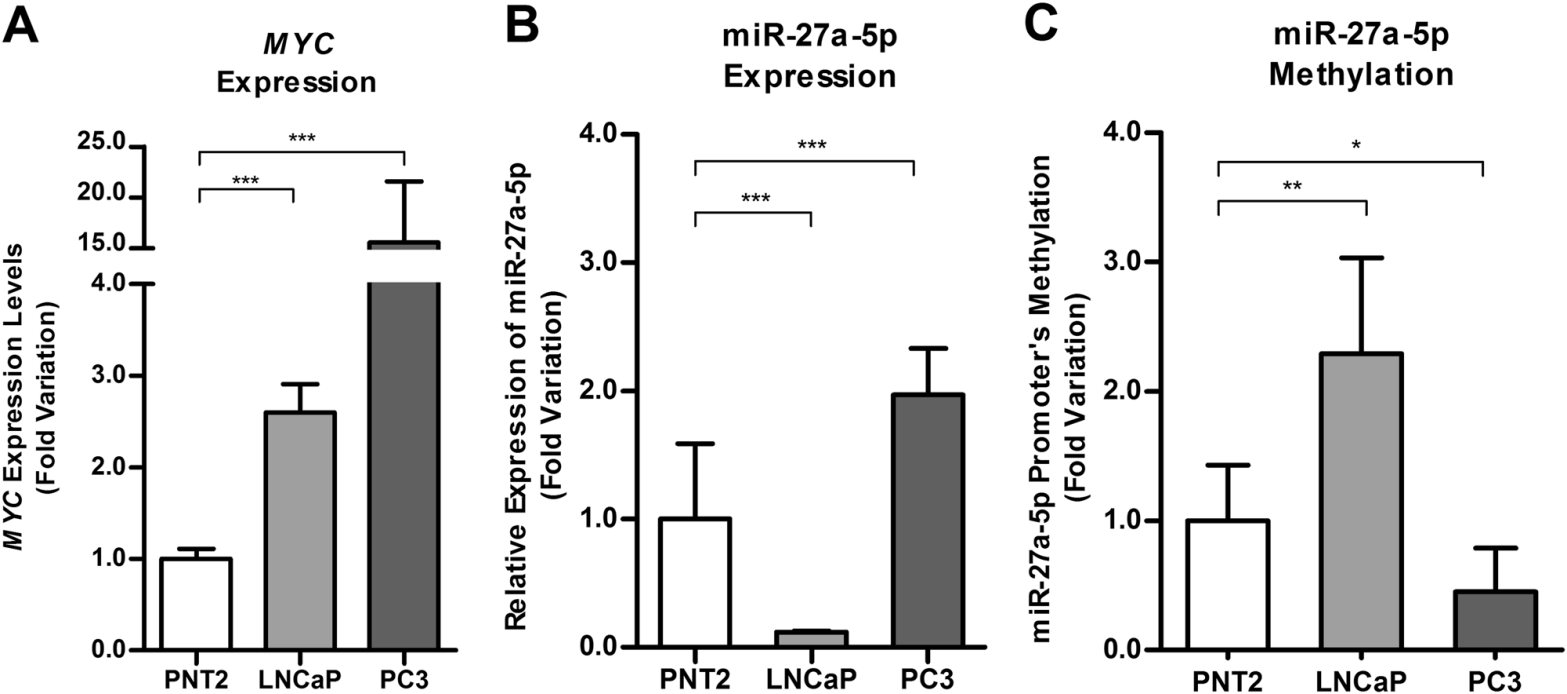
Expression of *MYC* in PCa cell lines (**a**); Expression (**b**) and methylation (**c**) of miR-27a-5p in PCa cell lines. Results are displayed after normalization to PNT2

PCa cell lines, LNCaP and PC3, displayed higher *MYC* expression levels in comparison with PNT2 (*P < *0.001). In PC3 cells, *MYC* expression levels were 15 times higher than in PNT2 cells (Fig. 4a).

Significant differences were found for miR-27a-5p expression and promoter methylation levels in LNCaP and PC3 cells compared to PNT2 (Fig. 4b, c). Indeed, LNCaP cells showed lower miRNA transcript levels (*P < *0.001) and higher miR-27a-5p promoter methylation levels (*P* = 0.004), whereas in PC3 cell line, higher miR-27a-5p expression levels (*P < *0.001) and lower methylation levels (*P = *0.019) were apparent.

### Impact of 5-AzaA-CdR exposure in PCa cell lines

**Fig. 5:**
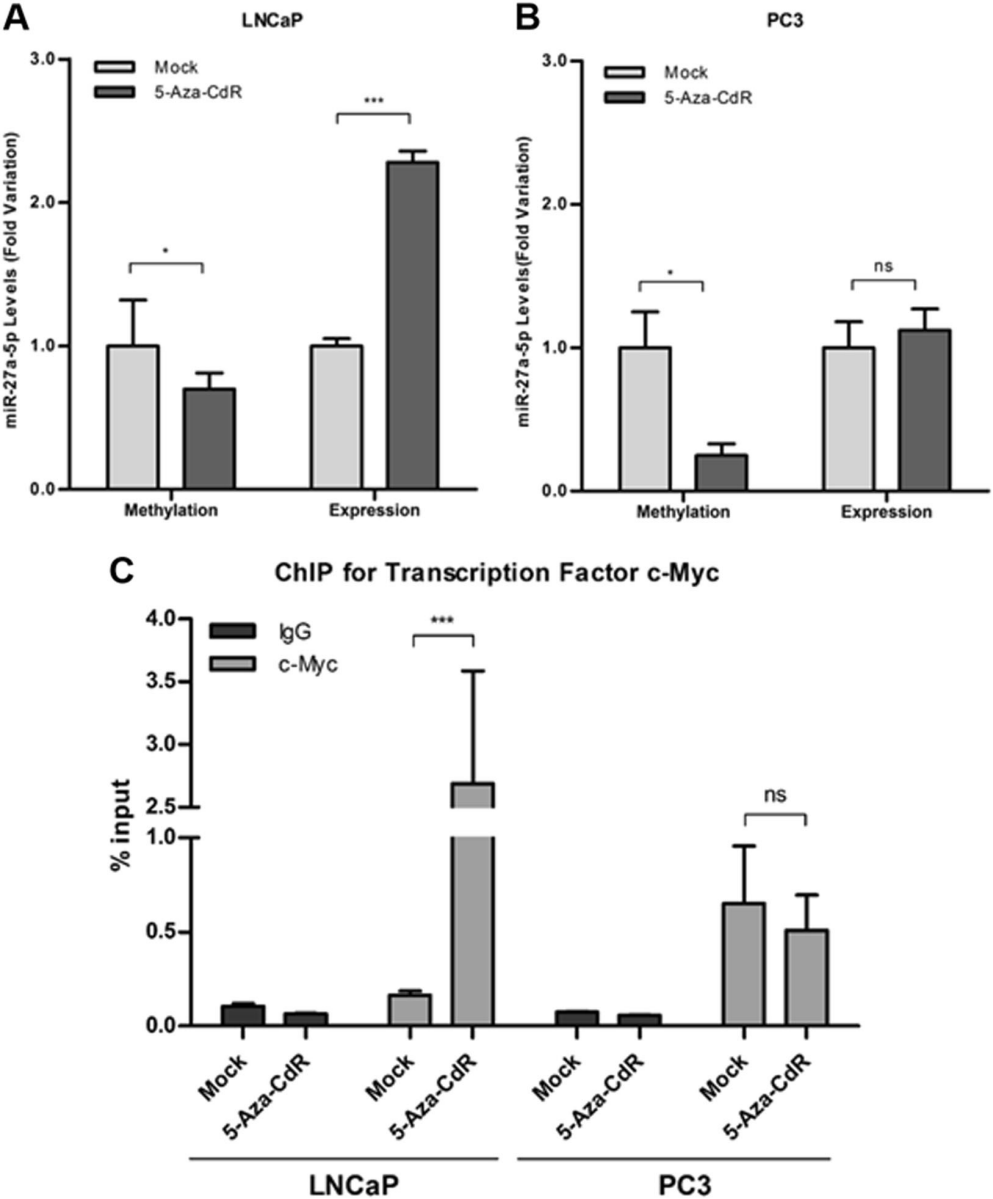
Methylation and expression levels of in LNCaP (**a**) and in PC3 (**b**) upon 5-Aza-CdR treatment (results are displayed after normalization to mock). Real-time PCR analysis of c-Myc chromatin immunoprecipitation at miR-27a-5p promoter region in LNCaP and PC3 cell lines before and after 5-Aza-CdR exposure (**c**)

To confirm whether miR-27a-5p expression was regulated by promoter methylation, PCa cell lines were exposed to 5-aza-2'deoxycytidine (5-Aza-CdR) (Fig. 5a, b), and a statistically significant reduction of miR-27a-5p promoter methylation levels was observed in both PCa cell lines (*P < *0.05). However, significantly higher miR-27a-5p re-expression (50% increase) was only observed in LNCaP cells (*P < *0.001).

### MicroRNA-27a-5p regulation by *MYC* signaling

In silico analysis for putative c-Myc-binding sites within miR-27a-5p promoter region was performed. c-Myc is known to bind to the canonical E-box sequence CACGTG^17^ and we identified one putative-binding site matching these sequence in miR-27a-5p promoter region. c-Myc regulation of miR-27a-5p expression was assessed in LNCaP cells (hypermethylated at miR-27a-5p’s promoter) and PC3 (hypomethylated at miR-27a-5p’s promoter) by ChIP (chromatin immunoprecipitation) and the results are depicted in Fig. 5c. A significant increase (*P* < 0.001) of c-Myc binding at miR-27a-5p promoter region was apparent in LNCaP 5-Aza-CdR-treated cells, whereas low c-Myc amount was found in mock cells. Conversely, in PC3, c-Myc enrichment was found at miR-27a-5p promoter both in mock and 5-Aza-CdR exposed PC3 cells. Therefore, our data suggest that miR-27a-5p is regulated by c-Myc depending on the methylation status of its promoter.

### MiR-27a-5p attenuates malignant phenotype in PCa cells

MiR-27a-5p mimic and anti-miR were transfected into LNCaP (cell line with the lowest miRNA expression) and PC3 (cell line with the highest miRNA expression) cells, respectively.

**Fig. 6:**
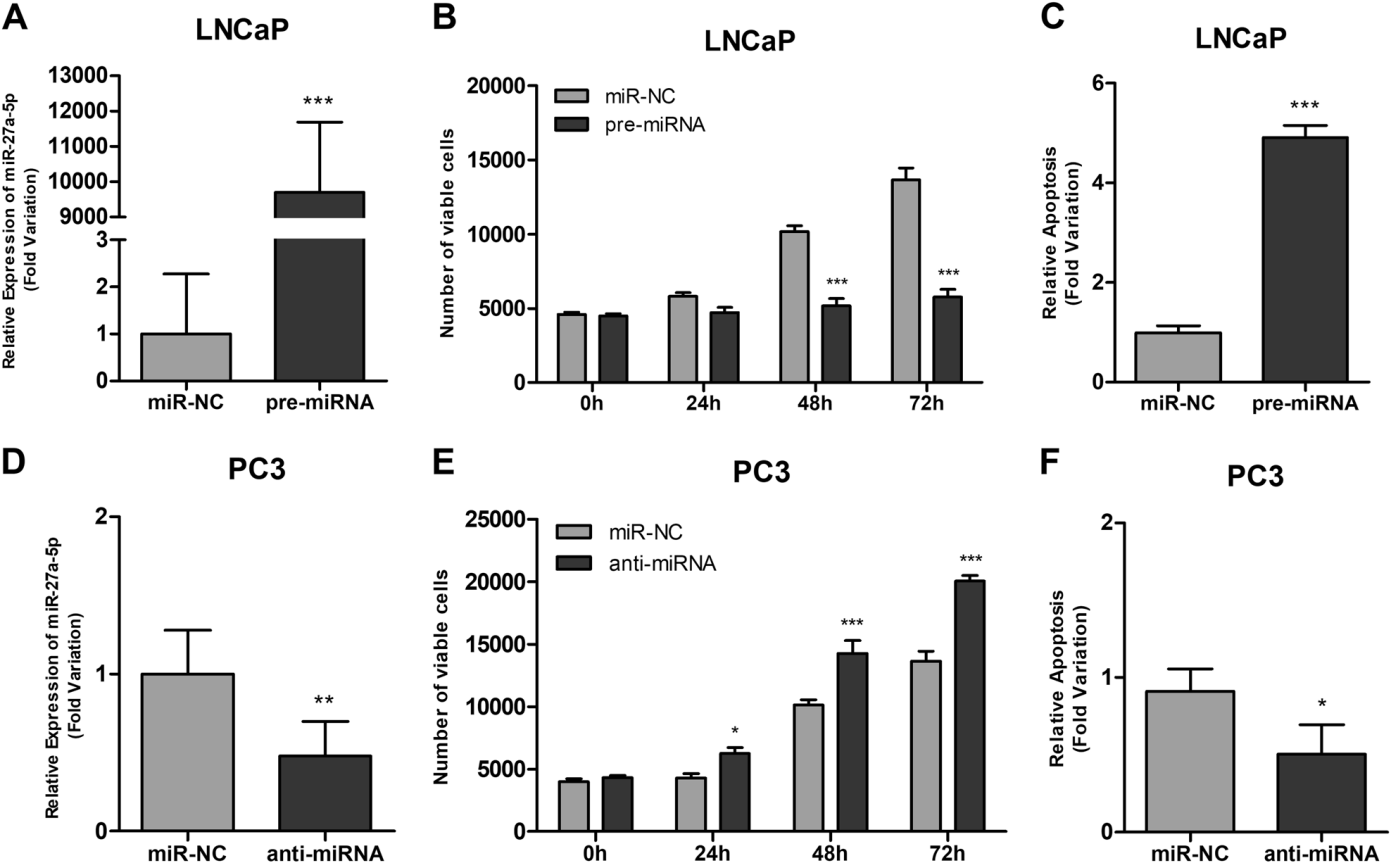
Confirmation of miR-27a-5p expression levels by RT-qPCR. Overexpression in post-transfected LNCaP cells with miR-27a-5p vs. miR-NC mimics and miR-27a-5p downregulation after transfection with anti-miR-27a-5p vs. anti-miR-NC in PC3, respectively (**a**, **d**). Cell viability measured by MTT assay at different time points (**b**,** e**) and apoptosis evaluation 72 h-post-transfection (**c**, **f**) for LNCaP and PC3, respectively

LNCaP transfection efficiency was confirmed by quantitative reverse transcription PCR (qRT-PCR) 72 h (*P* < 0.001) (Fig. 6a). MiR-27a-5p overexpression significantly reduced viability at 48 h (49%, *P* < 0.001) and 72 h (58%, *P* < 0.001) (Fig. 6b) and significantly increased apoptosis 72 h after transfection (about four times (*P* < 0.001) (Fig. 6c).

Conversely, in PC3, a 50% decrease in miR-27a-5p expression levels was achieved 72 h after anti-miR transfection (*P* < 0.01) (Fig. 6d). PC3 anti-miR-27a-5p transfected cells showed significant increased cell viability (32%) (*P* < 0.001) (Fig. 6e), whereas apoptosis was decreased by 50% (*P* < 0.05) (Fig. 6f).

### MicroRNA-27a-5p forced expression mimics *MYC* knockdown in PC3 cell lines

**Fig. 7:**
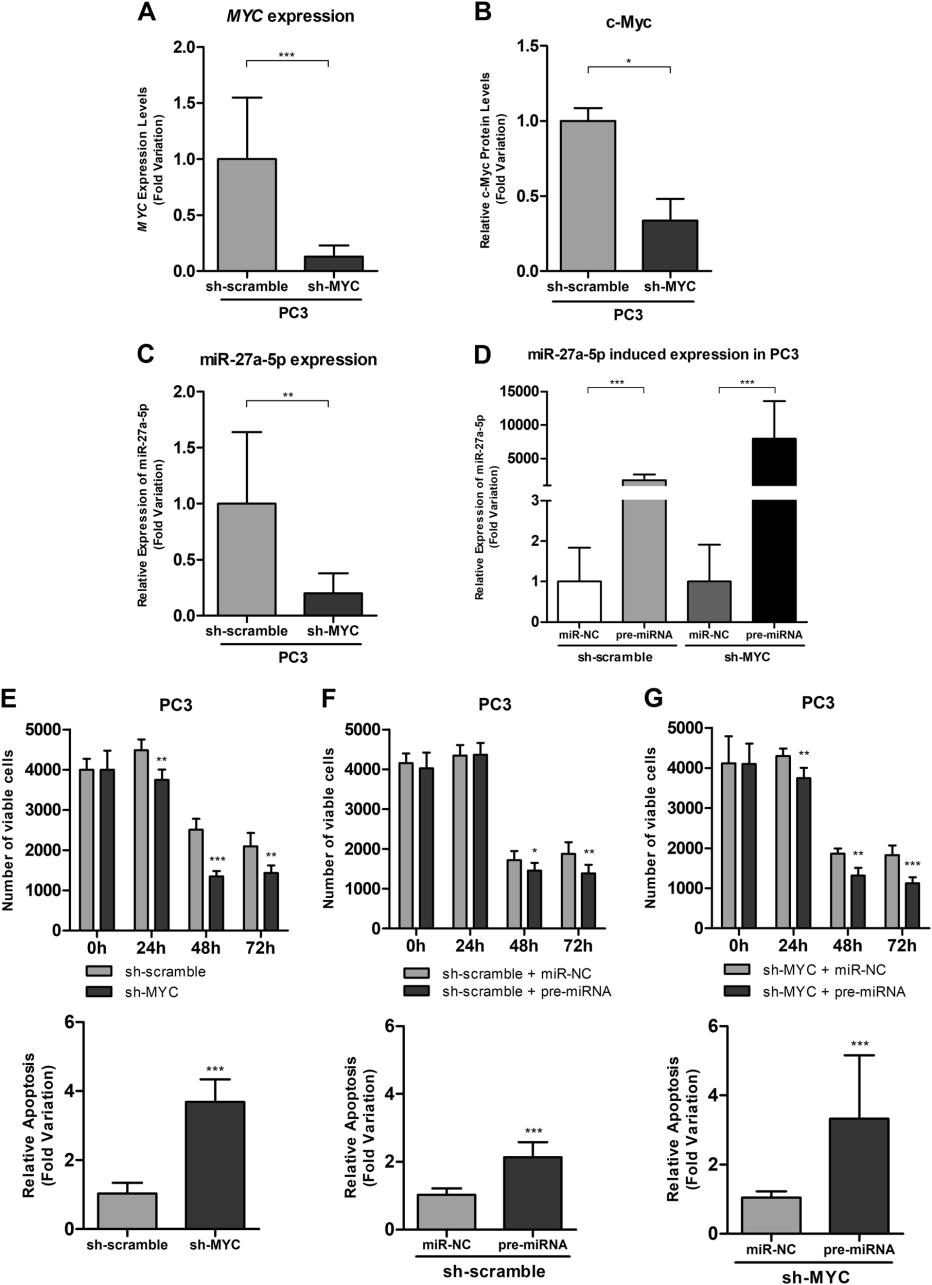
*MYC* expression (**a**), c-Myc protein levels (**b**), and miR-27a-5p expression after *MYC* knockdown in PC3 cell lines (**c**); Assessment of pre-miR-27a-5p transfection efficiency in PC3 scramble and Sh-MYC (**d**); Phenotypic impact (viability and apoptosis) of *MYC* knockdown (**e**), pre-miR -27a-5p transfection (**f**) and both (**g**) in PC3 cell line

Effective *MYC* silencing was achieved in PC3 cells, confirmed at mRNA (87%, *P* < 0.001) and protein level (50%, *P* < 0.05) (Fig. 7a, b, respectively). Furthermore, *MYC* knockdown attenuated the malignant phenotype with a statistically significant reduction of cell viability, more evident at 48 h (54%, *P* < 0.001), and an increase of apoptosis, at 72 h (about three times, *P < *0.001) (Fig. 7e).

After *MYC* silencing we found a significant reduction in miR-27a-5p transcript levels (80%, *P* < 0.01) (Fig. 7c). MiR-27a-5p mimics was transfected in sh-scramble and sh-MYC PC3 cell line and transfection efficiencies were confirmed by RT-qPCR (Fig. 6d). Forced miR-27a-5p expression in sh-scramble PC-3 cells caused an inhibitory effect on cell viability more evident at 72 h (32%; *P = *0.003) and increased apoptosis (1.92 times; *P < *0.001) also at 72 h’ post-transfection (Fig. 7f). In sh-MYC PC-3 cells transfected with miR-27a-5p, a significant reduction in number of viable cells was found, particularly at 72 h (40%; *P < *0.001) and a 2.94-fold increase in apoptosis (*P < *0.001) was also apparent at the same time point (Fig. 7g).

### Establishment of miR-27a-5p putative targets

**Fig. 8:**
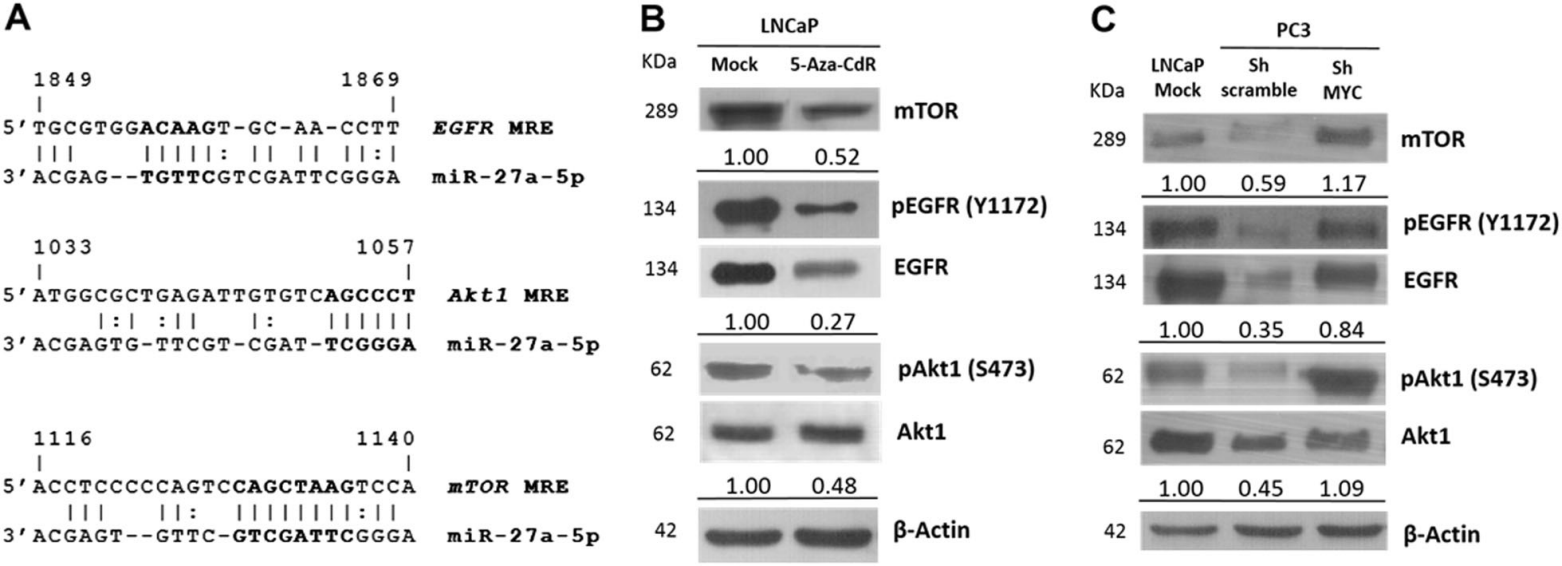
Identification of predicted miRNA response element (MRE) using in silico screening methods (**a**); Levels of EGFR/Akt1/mTOR signaling axis proteins’ in LNCaP mock and 5-Aza-CdR-treated cell lines (**b**) and in PC3 Sh-scramble and Sh-MYC (**c**) by western blot analysis: mTOR 289 KDa, EGFR 134 KDa, Akt1 62 KDa, and β-Actin 42 KDa (numbers represent the average fold-change compared with LNCaP mock for three independent experiments)

In search for genes regulated by miR-27a-5p, we evaluated putative targets associated with cellular processes and pathways relevant in PCa. The epidermal growth factor receptor (EGFR) signaling pathway has been previously reported as a target of miR-27a-5p in head and neck squamous carcinoma cell lines^18^ and in silico analysis identified one predicted miRNA response element (MRE) at CDS of EGFR. Further analysis for more potential targets of miR-27a-5p led to the identification of Akt1 (Akt serine/threonine kinase 1) and mTOR (mechanistic target of rapamycin) within the EGFR signaling axis (Fig. 8a, Supplementary Table 1), which are also documented as frequently deregulated in PCa^19^.

Because increased miR-27a-5p transcript levels were found upon 5-Aza-CdR exposure in LNCaP cells, we looked for altered protein expression of miR-27a-5p putative targets. Furthermore, we also assessed those target’s protein levels in PC3 cells, since these have much higher miR-27a-5p levels compared to LNCaP. Thus, decreased expression of EGFR, Y1172 EGFR phosphorylated, S473 Akt1 phosphorylated and mTOR, was found in 5-Aza-CdR exposed LNCaP cells, compared to mock (Fig. 8b). Similarly, decreased protein levels were observed in PC3 (sh-scramble) in comparison with LNCaP.

Contrarily, in *MYC*-knockdown (sh-MYC) PC3 cells, miR-27a-5p downregulation further increased the EGFR/Akt1/mTOR signaling axis (Fig. 8c).

## Discussion

Prostate cancer remains one of the major health challenges due to its incidence rate worldwide, lack of accurate biomarkers and scarce information concerning its onset and progression^20–22^. Thus, a more in-depth understanding of molecular alterations underlying prostate carcinogenesis may help improve current diagnostic and therapeutic approaches. Abnormal activation of the *MYC* oncogene may occur through several distinct mechanisms and it is currently recognized as a major event in many cellular pathways leading to the development of various types of neoplasia, including PCa^23^. Indeed, the c-Myc transcription factor, which may act as a transcription activator or repressor, greatly contributes to neoplastic transformation, by targeting genes with critical functions in cell cycle, differentiation, growth, metabolism, protein synthesis, adhesion, migration, angiogenesis, and many other processes^24^. Recently, not only c-Myc was shown to regulate the transcription of several miRNA, but also several miRNAs were suggested to regulate c-Myc expression^8,25,26^. These data support the existence of a complex regulatory network established between *MYC* and several miRNAs, which tightly controls the expression levels of target genes in a normal cell but, once deregulated, may be critical for cancer development.

Herein, we aimed to identify novel miRNAs implicated in prostate carcinogenesis that might be regulated by c-Myc. In a set of primary PCa, PIN, and MNPT samples (cohort #1), we confirmed *MYC* overexpression in PCa, in accordance with its oncogenic role^27,28^. Additionally, in the same cohort, higher c-Myc protein levels were statistically associated with predictors of more aggressive disease (higher serum PSA and GS), again in line with previous findings^29^. Moreover, in PIN lesions, c-Myc protein levels were higher than in MNPT but lower than in PCa, which is consistent with its precursor lesion status in prostate carcinogenesis^30^.

Microarray analysis identified three miRs—miR-27a-5p, miR-570, and miR-1292—as overexpressed in *MYC* upregulated PCa samples, suggestive of possible targets of c-Myc regulation. However, miRNA candidate validation was only accomplished for miR-27a-5p, because no successful amplification was accomplished for miR-570 and miR-1292, probably due to the very low levels of those two miRNAs in prostatic tissues. This highlights the importance of microarray validation through different techniques, as microarray and RT-qPCR methodologies have different detection sensitivities^31^. Interestingly, miR-27a-5p is part of the miR-23a-27a-24-2 cluster, previously reported to be frequently overexpressed^32^ and targeted by c-Myc regulation in breast cancer^33^.

Surprisingly, when miR-27a-5p expression levels were assessed in the large series of clinically localized PCa (cohort #1), for validation purposes, significant downregulation was found in PCa compared to MNPT, suggesting a tumor suppressive role for this miRNA, contrarily to the data from microarray analysis. Because we have found aberrant promoter methylation to be associated with silencing of several miRNAs in PCa^14–16^, we hypothesized that this epigenetic mechanism might be responsible for miR-27a-5p downregulation. Indeed, significantly higher methylation levels were found in PCa and PIN lesions comparatively with MNPT, and a significant inverse correlation between expression and promoter methylation levels was disclosed. This pattern suggest tumor suppressive functions for miR-27a-5p, in agreement with previous findings in different cancer models^18,34^. Interestingly, miR-23a, another member of miR-23a-27a-24-2 cluster, was also found downregulated due to promoter methylation^35^. Intriguingly, high-*MYC* PCa samples that displayed higher miR-27a-5p expression levels in the array, depicted the lowest methylation levels. Hence, the discrepancy between results of microarray and the validation cohort may be due to the small number of samples used in the array, whose selection was based only in *MYC* transcript levels. Thus, it is possible that we have introduced a significant bias in miRNAs analysis, emphasizing the need to always validate array results in an independent series with a different method.

As previously stated, the oncogenic role of *MYC* in prostate cancer is well documented and its amplification is often associated with the emergence of CRPC phenotype^36,37^. We confirmed this finding in a cohort CRPC tissues (cohort #2), which displayed *MYC* upregulation, with higher *MYC* transcript levels compared with clinically localized PCa (cohort #1, data not shown). Interestingly, in the CRPC cohort, miR-27a-5p expression and promoter methylation levels followed opposite trends compared with primary PCa (cohort #1). These results suggest that miR-27a-5p regulation might be context dependent (PCa vs. CRPC), with a predominantly epigenetic regulation in hormone-naive tumors, whereas other mechanisms prevail in advanced, castration-resistant disease.

Remarkably, the results observed in primary PCa and CRPC tissues were paralleled by those of obtained in PCa cell lines. Although *MYC* upregulation was found both in LNCaP (androgen-sensitive) and PC3 (androgen-insensitive) cells, expression levels were much higher in PC3, which presents copy number gains at 8q24 chromosome, including *MYC* loci^38^. Moreover, miR-27a-5p expression and promoter methylation levels disclosed the same trends found in primary PCa (LNCaP) and CRPC (PC3). Interestingly, although exposure to 5-Aza-CdR decreased promoter methylation levels in both cells lines, only in LNCaP cell significant restored expression was disclosed, reinforcing that in more aggressive cancer cells DNA methylation is not the main miR-27a-5p expression regulatory mechanism. In silico analysis identified putative c-Myc-binding sites at miR-27a-5p promoter region and ChIP assay results strongly suggested that miR-27a-5p’s regulation by c-Myc only occurs in the absence of promoter methylation. This is likely due to conformational modifications induced by DNA methylation in chromatin framework, preventing c-Myc binding at miR-27a-5p promoter region^39,40^.

These findings led us to evaluate the biological role of miR-27a-5p in PCa cells, using two complementary strategies. We found that forced miR-27a-5p expression in LNCaP cells attenuated the malignant phenotype, whereas anti-miR-27a-5p transfection in PC3 cells significantly enhanced the growth rate, thus indicating a growth-suppressive function for miR-27a-5p. We further verified that *MYC* knockdown in PC3 cells caused miR-27a-5p downregulation, supporting miR-27a-5p as a putative target of c-Myc regulation. Importantly, as expected, *MYC* knockdown mitigated PC3 malignant phenotype. The phenotypic impact of *MYC* knockdown was mimicked by miR-27a-5p ectopic expression (diminished viability and increased apoptosis) in PC3 cells. Indeed, in PC3 cells, although *MYC* positively regulates miR-27a-5p, the phenotypic impact of *MYC* silencing is different from miR-27a-5p knockdown, suggesting opposite roles for these molecules. Thus, while *MYC* plays an oncogenic role in prostate cancer, our results support a tumor suppressive function for miR-27a-5p in this cancer model.

Moreover, in *MYC*-knockdown PC3 cells, miR-27a-5p downregulation associated with increased EGFR/Akt1/mTOR oncogenic signaling axis, whereas 5-Aza-CdR-treated LNCaP cells with restored miR-27a-5p displayed EGFR/Akt1/mTOR pathway downregulation. EGFR signaling axis is deregulated in various solid tumors, including prostate cancer^41,42^, being commonly upregulated and fostering cancer cell growth^43^. Recently, *TOP2A*, *MELK*, and *CENPF* were also reported to be putative targets of miR-27a-5p and have been implicated in prostate cancer progression^34^. Indeed, *TOP2A* increased expression correlated with poor PCa prognosis^44^, *MELK* was found to be upregulated in advanced PCa^45^ and *CENPF* overexpression was shown to drive metastasis development during prostate cancer^46^. Remarkably, we found that lower miR-27a-5p expression levels associated with development of metastasis in the CRPC cohort (Supplementary Fig. 3). These data from primary tumors further supports the tumor suppressive function earlier suggested for miR-27a-5p by in vitro findings.

Considering these data, we propose that miR-27a-5p role in PCa depends on the stage of disease. In normal prostate epithelial cells, miR-27a-5p promoter in not hypermethylated and its expression is regulated by c-Myc, constituting a negative feedback loop that counteracts *MYC* signaling, eventually as a similar mechanism to oncogene-induced senescence. Then, at the earliest steps of neoplastic transformation, miR-27a-5p promoter gradually acquires methylation and its expression is silenced. This abolishes the *MYC* feedback loop and stimulates cell proliferation and survival, contributing to the emergence of PIN (which consists of neoplastic cells accumulate in glands, with preserved architecture, due to excessive proliferation and impaired cell death) and, subsequently, of invasive carcinoma. As PCa evolves, locus-specific hypermethylation is accompanied by global expansion of DNA hypomethylation, causing chromosome instability^47^, which promotes disease progression and metastatic spread. Thus, in CRPC, the miR-27a-5p promoter becomes hypomethylated, allowing for c-Myc to resume its regulatory role and leading to increased miR-27a-5p expression. However, at this stage, miR-27a-5p increased expression is no longer sufficient to halt PCa progression as cancer cells have acquired many genetic and epigenetic alterations, which concur to the fatal evolution of the disease. Although this is a rather speculative hypothesis (Supplementary Fig. 4), the data presented here fully support it and provides a framework for subsequent research in this field.

In conclusion, our study provides further insight into miRNAs’ deregulation in PCa. Specifically, this is the first study reporting the interplay between two independent mechanisms, aberrant promoter methylation and *MYC* signaling, in the regulation of miR-27a-5p in prostate cancer. Our results further emphasize that the role of miRNA deregulation in neoplastic transformation and progression is highly context-dependent, even in the same cancer model.

## Materials and methods

### Patients and sample collection

Primary tumors from 198 patients harboring clinically localized prostate carcinoma (PCa) were prospectively collected after diagnosis and primary treatment with radical prostatectomy, at Portuguese Oncology Institute of Porto (IPO-Porto). In 37 cases, PIN lesions were available and were included in this study. A set of 24 castration-resistant PCa collected from patient that underwent transurethral resection of prostate due to urinary obstruction were also included. Ten morphologically normal prostate tissues (MNPT) were collected from prostatic peripheral zone of bladder cancer patients submitted to cystoprostatectomy, to serve as controls. Histological slides from formalin-fixed paraffin-embedded tissue fragments were obtained from the surgical specimens and assessed for Gleason score and TNM stage. Relevant clinical data was collected from clinical charts. Informed consent was obtained from all participants, according to institutional regulations. This study was approved by the institutional review board [Comissão de Ética para a Saúde-(CES-IPOFG-EPE 205/2013)] of IPO Porto.

### Total RNA extraction

Total RNA from clinical samples and cell lines was obtained by suspension in TRIzol^®^ reagent and total RNA was purified from the aqueous phase of TRIzol^®^ extract using PureLink^™^ RNA Mini Kit (Invitrogen, Carlsbad, CA, USA) following manufacturer's recommendations.

### *MYC* expression

For each sample, first strand synthesis was performed using the High-Capacity cDNA Reverse Transcription Kit (Applied Biosystems, Foster City, CA, USA). *MYC* Expression levels were quantified by RT-qPCR using TaqMan® Universal PCR Master Mix and *MYC* TaqMan^®^ Gene Expression Assay (Hs00153408_m1) and *GUSβ* (Hs99999908_m1) (Applied Biosystems) was used as a reference gene for normalization. Ratios were then multiplied by 1000 for easier tabulation.

### Immunohistochemistry (IHC)

c-Myc expression was assessed by immunohistochemistry in FFPE sections using the anti-c-Myc rabbit monoclonal antibody (Abcam, Cambridge, MA, USA) at 1:100 dilution with Novolink™ Polymer Detection System (Leica Biosystems, Germany). A Burkitt’s lymphoma case served as positive control. An experienced pathologist evaluated c-Myc immunoexpression in an optical microscope, using a previously published score^48^. Accordingly, cases with *a*+1 score were considered negative for c-Myc overexpression, and cases with *a*+2 or +3 score were considered as positive for c-Myc overexpression.

### MicroRNAs global expression

Total mRNA was hybridized to one-color Agilent human miRNA platform SurePrint G3 unrestricted miRNA microarray, 8 × 60 K (Agilent Technologies). A total of 100 ng of total RNA was hybridized using miRNA complete labeling and the Hyb Kit (Agilent Technologies), according to the manufacturer’s instructions. The reactions followed a 2-step preparation, represented by dephosphorylation and denaturation of the total RNA incorporated with Cy3 fluorochrome by the T4 ligase. The next steps included standard washing procedures and hybridization with microarrays slides. After hybridization, the slides were scanned using a Microarray Scanner with Surescan High-Resolution Technology (Agilent Technologies). Data quantification and quality control were performed using the Feature Extraction (FE) software version 11.0.1.1 (Agilent Technologies). Expression data were loaded into R environment version 3.3.2^49^. Background adjustment was done by subtracting median background values from the median expression values obtained by FE, and data were subsequently log-transformed. Finally, all the data distribution was normalized by quantile function using the aroma-light bioconductor package^50^. Differentially expressed miRNAs were identified using the *T*-test by multtest package^51^. MiRNAs were considered to be of interest if *P*-values ≤ 0.01, fold-change ≥ 2.0 and receiver operating characteristic (ROC) curve with good predictive performance (AUC ≥ 0.9). ROC analysis was performed using ROCR package^52^. Hierarchical clustering of differentially expressed genes was performed using average linkage criterion and Euclidean distance as a metric^53^.

### MicroRNAs expression assay

Reverse transcription (RT) was performed to a total of 350 ng using TaqMan MicroRNA Reverse Transcription Kit and Megaplex RT human pool A v2.1 and B v3.0 (Applied Biosystems) according to the manufacturer’s instructions.

Quantitative real-time PCR (RT-qPCR) was performed using TaqMan small RNA Assays for miR-27a-5p (assay ID: 0004501) and TaqMan Universal PCR Master Mix II no UNG (2×) (Applied Biosystems), according to the recommended protocol. For each sample, miRNA expression was normalized to the endogenous control RNU48 (assay ID: 001006). Results were then multiplied by 1000 for easier tabulation.

### DNA extraction and bisulfite modification and quantitative methylation-specific PCR

One-thousand nanogram of DNA was extracted from all clinical samples and cell lines using phenol–chloroform method. The bisulfite modification was accomplished using EZ DNA Methylation-Gold™ Kit (Zymo Research, Orange, CA, USA), that integrates DNA denaturation and bisulfite conversion processes into one-step, according to the recommended protocol.

Quantitative methylation-specific PCR (qMSP) assays was performed using AmpliTaq Gold® DNA polymerase (Applied Biosystems), according to the recommended protocol. Sequence-specific primers and a Taqman probe used in this study were synthesized by Sigma-Aldrich (Sigma-Aldrich, CO., St. Louis, MO, USA) (Supplementary Table 2). In each sample, the miRNA-27a-5p DNA methylation status was normalized to the endogenous control *β-Actin*. Results were then multiplied by 1000 for easier tabulation.

### Prostate cell lines

Three prostate cell lines were obtained from ATCC—American Type Culture and included in the study: PNT2 (benign cell line), LNCaP (hormone-sensitive), and PC3 (castration-resistant). All cell lines were cultured using recommended medium supplemented with 10% of fetal bovine serum and 1% of penicillin–streptomycin (GIBCO, Invitrogen, Carlsbad, CA, USA) and maintained at 37 °C and 5% CO_2_ in a humidified chamber. All PCa cell lines were routinely tested for *Mycoplasma* spp. contamination (PCR Mycoplasma Detection Set, Clontech Laboratories Inc., Mountain View, CA, USA).

### Cell lines treatment with epigenetic-modulating drug

To reverse DNA methylation effect in the cell lines, we used 1 μM of the DNA methyltransferases inhibitor 5-aza-2-deoxycytidine (5-Aza-CdR; Sigma-Aldrich). The cell lines were grown until 20 to 30% of confluence and, then, medium containing the drug was added. On day 4, the cells were harvested by trypsinization. Pellets were stored for DNA and RNA extraction.

### In silico analysis

In silico analysis was performed to calculate the probability of c-Myc to bind to the promoter of gene where validated miRNA are inserted, based on a recently reported c-Myc-binding sequence and its respective binding matrix^54^. MiRNA promoter sequence were obtained from *Genome Browser* database (MIR27A: 19p13.12) and the number of transcription factor binding sites was retrieved with the help of *ConSite* web-based tool, after the alignment between miRNA promoter sequence and c-Myc-binding sequence. Additionally, RNA22 tool^55^ was used to predict miRNA target sites in the mRNA sequence of EGFR, Akt1, and mTOR.

### Chromatin immunoprecipitation and RT-qPCR

Cells were crosslinked with formaldehyde (37%) and chromatin was immunoprecipitated using the iDeal Chip-seq Kit for Transcription Factors (Diagenode, Belgium), according to the recommended protocol. Rabbit monoclonal antibody specific for c-Myc (Abcam®) was used to immunoprecipitate chromatin fragments and rabbit IgG antibody was used as negative control. Real-time PCR was performed using NZYSpeedy qPCR Green Master Mix (NZYTech, Portugal). Sequences of primers used to amplify ChIP samples were: primer forward 5′-TGCTTGGCCTGAAATTCTTAG-3′ and primer reverse 5′-ACCAGGGCAAGATACAGGA-3′. To analyze the results, the percentage input method was used.

### *MYC* gene silencing

*MYC* gene silencing in PC3 cells was achieved through the use of particles carrying the pGIPZ lentiviral vector containing a short hairpin RNA (shRNA) sequence targeting *MYC* (Applied Biosystems). As a negative control, one scrambled siRNA (sh-scramble RNA) sequence was used. After transduction, stable clones with shRNA were selected with Puromycin dihydrochloride (cat. 631306, Clontech Laboratories Inc.) at a final concentration of 4 μg/mL.

### MicroRNA transfection

MiRNA-27a-5p was transiently transfected in LNCaP, PC3 sh-scramble, and PC3 sh-MYC with Pre-miR^TM^ miRNA Precursor (has-miR-27a-5p, AM17100, Applied Biosystems) and in PC3 with Anti-miR^TM^ miRNA Inhibitor (has-miR-27a-5p, AM17000, Applied Biosystems), each at 50 nM. A miRNA negative control was used as control in all experiments (miR-NC, AM17010, Applied Biosystems). Transfection were performed using Oligofectamine (Invitrogen) and detailed procedure was described elsewhere^56^.

### Phenotypic assays

To evaluate the impact of in vitro transfection of miR-27a-5p in LNCaP and PC3, 3-(4,5-dimethylthiazol-2-yl)-2,5-diphenyltetrazolium (MTT; Sigma-Aldrich) assay was performed. Apoptosis was assessed using the APOPercentage™ kit (Biocolor Ltd., Belfast, Northern Ireland, UK). The procedures followed the published report^56^.

### Protein extraction and quantification

Nuclear protein was extracted from PC3 cells using performed using Nuclear Extract Kit (Active Motif) and total protein was extracted from LNCaP cell lysates using the radioimmuno precipitation assay (RIPA) (Santa Cruz Biotechnology Inc., Santa Cruz, CA, USA) and subsequently quantified using a Pierce BCA Protein Assay Kit (Applied Biosystems), according to the manufacturer's instructions.

### Western blot

Briefly, 30 µg of protein from each sample were separated using 10% sodium dodecyl sulfate polyacrylamide gel, for further electrophoresis (SDS–PAGE) at 120 V and subsequently blotted onto 0.2 μm nitrocellulose membranes (Bio-Rad Laboratories Inc., Hercules, CA, USA). After that, membranes were blocked with a 5% non-fat dry milk solution in TBS-T and then incubated with antibody. To ascertain equal loading of protein, the membranes were incubated with an endogenous control antibody. All antibodies used are listed in Supplementary Table 3. Protein band intensities were determined using ImageJ (Wayne Rasband software from National Institute of Health), by comparing the protein band intensity with the loading control (LMNB1 in nuclear protein extract and β-Actin in total protein extract).

### Statistical analysis

Unless otherwise stated, experiments were performed in triplicates. The Shapiro–Wilk’s W test allowed for the examination of the appropriateness of a normal distribution assumption for each of the parameters (data not shown). Comparisons between two groups were then performed using non-parametric Mann–Whitney *U*-test. *P*-values were considered statistically significant if <0.05. Correlation between miRNAs’ expression and methylation were measured by the Spearman correlation coefficient (*r*) test. Data are presented as median ± interquartile range for tissue analysis and mean of three independent experiments ± SD for cell line analysis. Significance is shown vs. the respective control and depicted as follows: *P < 0 .05, **P < 0.01, ***P < 0.001 and ns—non-significant.

Statistical analysis was performed using SPSS 20.0 for Mac (IBM-SPSS Inc., Chicago, IL, USA) and graphs were built using GraphPad Prism 5.0 software for Mac (GraphPad Software Inc., La Jolla, CA, USA).

## Electronic supplementary material

Supplementary Figure 1

Supplementary Figure 2

Supplementary Figure 3

Supplementary Figure 4

Supplementary Figure legends

Supplementary Tables
