# Supplementary figures and images for: MicroRNA-27a-5p regulation by promoter methylation and MYC signaling in prostate carcinogenesis
Source: Cell Death Dis. 2018 Feb 7;9(2):167. doi: 10.1038/s41419-017-0241-y (PMC5833437; doi:10.1038/s41419-017-0241-y)

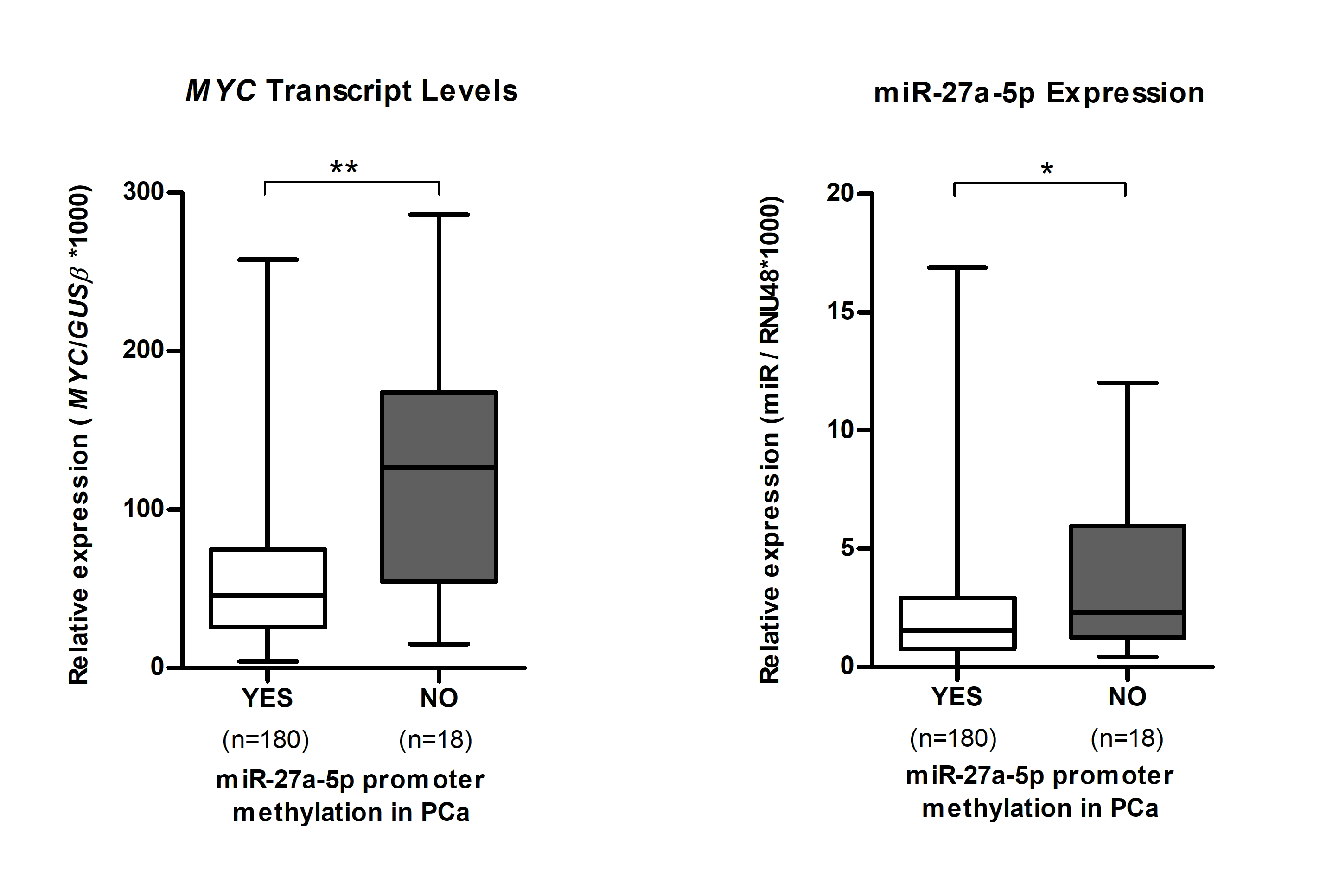

Supplement: Supplementary file 2 — Supplementary Figure 2 [file 41419_2017_241_MOESM2_ESM.tif]

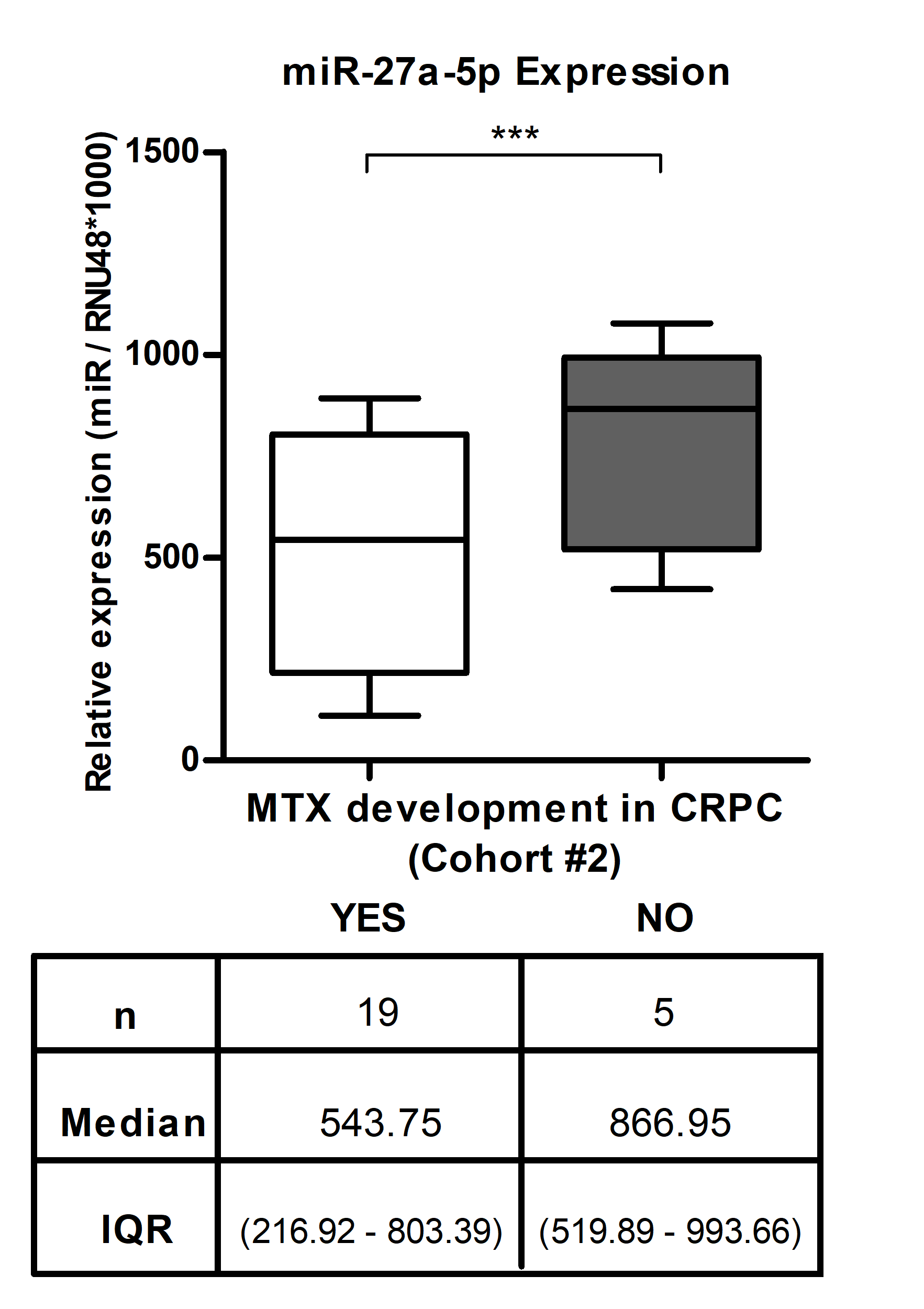

Supplement: Supplementary file 3 — Supplementary Figure 3 [file 41419_2017_241_MOESM3_ESM.tif]
