# Supplementary material for: MicroRNA-27a-5p regulation by promoter methylation and MYC signaling in prostate carcinogenesis
Source: Cell Death Dis. 2018 Feb 7;9(2):167. doi: 10.1038/s41419-017-0241-y (PMC5833437; doi:10.1038/s41419-017-0241-y)
Supplement: Supplementary file 5 — Supplementary Figure legends [file 41419_2017_241_MOESM5_ESM.docx]

**Supplementary Figure Legends**

**Supplementary Figure 1** Heatmap resulting from the miRNA microarray performed with total RNA extracted from PCa cases with high and low levels of *MYC* transcript. The selected miRNAs candidates were evidenced by the black arrows.

**Supplementary Figure 2** *MYC* and miRNA-27a-5p expression levels according with miR-27a-5p promoter methylation in PCa cases.

**Supplementary Figure 3** Expression levels of miRNA-27a-5p according with metastasis development in CRPC (Cohort #2).

**Supplementary Figure 4** Proposed model for miR-27a-5p’s regulation in Prostate Cancer.
