# Supplementary material for: MicroRNA-27a-5p regulation by promoter methylation and MYC signaling in prostate carcinogenesis
Source: Cell Death Dis. 2018 Feb 7;9(2):167. doi: 10.1038/s41419-017-0241-y (PMC5833437; doi:10.1038/s41419-017-0241-y)
Supplement: Supplementary file 6 — Supplementary Tables [file 41419_2017_241_MOESM6_ESM.docx]

**Supplementary Table 1** miRNA pre-computed static target predictions based on the RNA22 tool.

| **hsa-miR-27a-5p**  **miRNA Response Elements (MRE)** | | | | |
| --- | --- | --- | --- | --- |
| **Target** | **cDNA region** | **Left most position of predicted target site** | **Folding energy (Kcal/mol)** | ***P* value** |
| ***EGFR*** | CDS | 1849 | -12.20 | 0.00777 |
| ***AKT1*** | CDS | 1033 | -13.90 | 0.0135 |
| ***mTOR*** | CDS | 1116 | -19.00 | 0.0381 |

**Supplementary Table 2** TaqMan probe and primer sequence, amplicons, locations and annealing temperatures for qMSP.

| **MiR-27a-5p (Family Name: *MIR27*)** | |
| --- | --- |
| **Forward Sequence** | ^5’^TGTATTTTAGTCGTGGCGATA^3’^ |
| **Probe Sequence** | ^5’^(6-FAM)AGAGATGGGGTATTGTCGTATTGC(BHQ-1)^3’^ |
| **Reverse Sequence** | ^5’^ATAACGACTCACGCCTATAATC^3’^ |
| **Amplicon Size (bp)** | 172 |
| **Location (bp upstream TSS)** | 1222 |
| **Annealing Temp (^o^C)** | 60 |
| ***β-Actin*** ^(Eads^ *^et al^*^., 2001)^ | |
| **Forward Sequence** | ^5’^TGGTGATGGAGGAGGTTTAGTAAGT^3’^ |
| **Probe Sequence** | ^5’^(6-FAM)ACCACCACCCAACACACAATAACAAACACA(TAMRA)^3’^ |
| **Reverse Sequence** | ^5’^ACCAATAAAACCTACTCCTCCCTTAA^3’^ |
| **Amplicon Size (bp)** | 132 |
| **Location (bp upstream TSS)** | 1599 |
| **Annealing Temp (^o^C)** | 60 |

**Supplementary Table 3** All antibodies used in Western blot.

| **Antibodies** | **Vendor** | **Catalog number** |
| --- | --- | --- |
| Anti-c-Myc antibody | Abcam, Cambridge, MA, USA | ab32072 |
| Anti-LMNB1 antibody | Cell Signaling Technology, Danvers, MA, USA | 12586S |
| Anti-EGFR antibody | Kinexus Inc., Vancouver, Canada | AB-NK052-4 |
| Anti-EGFR phospholylated Y1172 antibody | ProSci Inc., Poway, CA, USA | XBP-4085 |
| Anti-Akt1antibody | Santa Cruz Biotechnology, Santa Cruz, CA, USA | sc-5298 |
| Anti-Akt1 phosphorylated S473 antibody | EMD Millipore, Temecula, CA, USA | 05-1003 |
| Anti-mTOR antibody | Cell Signaling Technology, Danvers, MA, USA | 2972 |
| Anti-β-Actin antibody | Sigma-Aldrich, CO., St Louis, MO | A5316 |
